# Exploring the meaning of caring amongst student midwives, professional midwives and educators in Tshwane, South Africa

**DOI:** 10.4102/phcfm.v7i1.894

**Published:** 2015-12-31

**Authors:** Mmajapi E.T. Masala-Chokwe, Tendani S. Ramukumba

**Affiliations:** 1 Adelaide Tambo School of Nursing Science, Tshwane University of Technology, South Africa

## Abstract

**Background:**

In spite of caring being the core of midwifery and nursing, there is a constant public outcry about uncaring behaviours of midwives towards clients. Local media reports and recent discussion in the health system have highlighted the concerns regarding caring behaviours of midwives. Thus, there is a need to compare the meaning of caring from the perspectives of student midwives, midwives and educators**.**

**Aims:**

The aim of this article was to determine the meaning of caring from the perspectives of the undergraduate student midwives, the professional midwives, and the educators teaching midwifery in Tshwane.

**Setting:**

The study was conducted at healthcare institutions which the undergraduate student midwives attended for work integrated learning and at nursing education institutions in Tshwane, South Africa.

**Methods:**

The strategy was qualitative and exploratory in nature. The population comprised student midwives, professional midwives and educators. Purposive sampling was done. Focus group discussions were held with student midwives and professional midwives, whilst the naïve sketch form was used as data-collection instrument for educators. Content data analysis was done. The total sample realised was 38.

**Results:**

The findings revealed that ‘caring’ was taken to mean being well conversant, upto-date and proficient in the field of work as well as considerate and respectful to others. The professional midwives indicated that they have seen colleagues demonstrate uncaring behaviour whilst educators emphasised respect as caring.

**Conclusion:**

The student midwives, professional midwives and educators described caring as being a competent nurse with compassion and respect for others.

## Abstrait

**Les connaissances des prestataires de soins communautaires sur les pratiques familiales clés dans une communauté rurale d'Afrique du Sud.**

**Contexte:**

Les interventions par des prestataires de soins communautaires dans le contexte de la gestion intégrée communautaire des maladies de l'enfance (GICME) peuvent avoir un effet positif sur la santé des enfants si les prestataires de soins ont suffisamment de connaissances sur les pratiques familiales clés.

**Lieu:**

L’étude a été menée dans les régions rurales de la Cote Ouest du Western Cape, en Afrique du Sud.

**Objectifs:**

L'objectif de cette étude était de déterminer les connaissances des prestataires de soins communautaires (PSC) sur cinq des 16 pratiques familiales clés de la GICME.

**Méthodes:**

Un plan de recherche descriptif et exploratoire avec approche quantitative a été utilisé. On a obtenu un taux de réponse de 95.2% de la population ciblée (*N* = 270). On a collecté les données au moyen d'un questionnaire auto-administré. On a utilisé une analyse statistique descriptive et différents tests statistiques…

**Résultats:**

Seuls 25 des répondants (10%) ont obtenu un score supérieur à 70% sur les questions du questionnaire basées sur les connaissances. Les connaissances des PSC étaient très faibles dans le domaine de la morbidité élevée et des taux de mortalité des enfants. Moins de 25% des répondants ont répondu correctement aux questions dans ces domaines clés (pneumonie [17%], tuberculose [13%], VIH/SIDA [9%] immunisation [3%] et recommandations pour un enfant fiévreux [21%]). On a trouvé des corrélations statistiquement importantes entre le score total d'un répondent et le plus haut niveau d’éducation atteint (*P* < 0.01), le niveau de formation en cours d'emploi (*P* < 0.01), assistance à un cours de formation de cinq jours sur la GICME (*P* < 0.01), et l'achèvement ultérieur d'un cours de rafraichissement (*P* < 0.01).

**Conclusion:**

Il faut offrit tous les ans des cours de rafraichissement sur la GICME pour améliorer les connaissances des PSC et la qualité des soins qu'ils donnent. Des cours de mise à jour réguliers pourraient permettre de développer les compétences.

## Introduction

No-one exists in isolation because humans are connected to one another by caring, an aspect which is important for human existence.^1^ When people are in vulnerable situations, such as being ill or pregnant, they express a strong need for care and caring.^1^ Nursing, which includes midwifery, is a profession of relationships. Therefore, the profession has the obligation to revisit the value central to the profession, namely caring.^1^

Caring is primary to nursing and midwifery. According to Fahrenwald et al.^2^ caring is demonstrated in nursing when the nurse or midwife expresses the five core values of the profession: human dignity, integrity, autonomy, altruism and social justice. Student midwives should observe caring behaviours by the educators and professional midwives as the custodians of and role models for caring. Furthermore, in order to build a strong caring profession, caring should be interlaced through the curriculum of nursing and midwifery training.

Teaching caring should include both the emotional and the physical aspects, so that all nursing and midwifery actions provided to patients are accompanied by empathy, respect, compassion.^2^ To effectively teach caring, educators and midwives should model the attributes of caring, value the students and believe in the students’ potential for growth.^2^ In addition, the professionals must feel good about themselves and confident in their roles; educators should excel in their subjects and midwives should be willing to become involved with the students.^3^

Nursing and midwifery do not always show outcomes of care in the same way as doctors’ curative acts of care do. Nursing outcomes are mostly based on the caring attributes experienced by those who are cared for. The attributes which help to identify specific caring behaviours during nursing and midwifery care are competence, conscience, confidence, commitment, comportment and creativity.^3^ Therefore, it is important for the nursing and midwifery professions to model caring so that the intended outcomes of interconnectedness are realised.^1^

Although caring is the core of midwifery, there is a constant public outcry about uncaring behaviours of midwives towards clients.^4^ Local media reports and recent discussion in the health system have highlighted the concerns regarding caring behaviours of midwives.^5,6^ In the past many institutions and literature raised awareness of and enormous concern about the negative midwife-client relationship; nonetheless the problem still exists in South Africa and internationally.^7^ Miller and Keane^8^ define caring as an emotional commitment to and willingness to act on behalf of persons with whom one has a special relationship. These include family members, relatives, friends and patients. In that relationship there is love, trust, compassion, sympathy and empathy.^7^

Flyn^9^ states that caring is unique to each individual and therefore the definition of caring will be dependent on the needs of the individual. One therefore cannot assume that caring will be defined the same by health professionals, student midwives, educators and patients.^10^ Perhaps the starting point therefore is to establish the meaning of caring as described by student midwives, professional midwives and educators.

Like other professions, midwifery in South Africa has experienced changes as a result of political, technological, health and disease specialisations.^11^ In addition, overexpansion of services is complicated by staff shortages, resulting in decreased contact time with patients whilst pressure regarding patient throughput increases.^11,12^ According to Watson,^13^ nursing in general has lost its humanness and caring; these have been replaced by machines, technology and the medicalisation of childbirth. Some authors^11,12,14^ claim that caring is further affected by the worsening conditions under which nurses and midwives work. Griffiths^14^ agrees with Corbin^15^ about the changing environment of nursing and how caring has been affected by staff shortage staff and the use of technology^16^; however, Griffiths suggests that in order to counteract these conditions, caring must constantly be nurtured in each individual on a continuous basis.

Many theorists have defined caring. Leininger^17^ defines professional caring as knowledge acquired through institutions used to assist, support and enable another individual to improve a health condition. Furthermore, caring behaviours can be categorised as belonging to the emotional or cognitive aspect or the instrumental or physical aspect of caring.^18^ The cognitive type of caring is based on assisting the recipient of caring in coping with the situation, whereas the instrumental type of caring focuses on the actions of alleviating suffering, for example giving analgesia to a patient.^18^

Notwithstanding the efforts by stakeholders to improve caring in the midwifery and nursing profession, there is still lack of caring in midwifery practice.^19,20,21^ The purpose of this study was to determine the meaning of caring from the perspectives of undergraduate student midwives, professional midwives working at hospitals and clinics, and educators teaching midwifery. The objectives of the study were to explore and describe the meaning of caring from the perspectives of student midwives, professional midwives and educators in midwifery clinical practice.

## Problem statement

The science of caring is changing and developing continuously. In addition, individuals’ views are altered by cultural change, diversity of philosophies and legal and political milieus. From the introduction it is clear that much has been written about nurses and caring but practically, caring seems to be lacking. Unpublished information gathered during debriefing sessions in class with student midwives revealed that they experienced uncaring behaviours from the educators and the professional midwives in clinical practice.^22^ In addition, they had different experiences and definitions of caring, although they belonged to the same institution and were at the same level of study.^23^ If educators instruct student midwives about caring, why do student midwives seem to lose their caring attributes when they start practising midwifery and nursing? In addition, how do educators define and express caring to student midwives? The uncaring behaviour of professional midwives observed in clinical practice is also a matter for concern.

Therefore, there is a need to explore the perception of caring amongst the three groups of participants in order to find a starting point for improving caring behaviours in midwifery clinical practice. As a result of the inconsistent caring experienced by student midwives, and the general public outcry regarding uncaring behaviours of professional midwives in clinical practice, the researcher identified a need to explore the meaning of caring from the perspective of student midwives, professional midwives and educators.

### Research question

The research question therefore is: what does caring mean from the perspective of the student midwives, professional midwives and educators?

### Significance of the study

Caring is the basis of the nursing and midwifery professions, therefore misconceptions regarding care or a lack thereof may affect patient care negatively. We intend to show that student midwives, professional midwives and educators ascribe different meanings to the concept of caring. If the meaning of caring is described as an activity and not as an emotional encounter, then the recipients of caring might not experience it as expected. Furthermore, the difference in meaning may have an impact on the type of care and caring imparted to patients, colleagues and the community. If caring is defined based on the cognitive aspect only, excluding the emotional and expressive aspect which is the core of caring, the profession might be missing an important aspect of caring as an affective skill. The importance of the affective, emotional aspect of caring cannot be underestimated as it is what the recipients of care expect when they encounter nursing and midwifery services.^23,24^

## Research methods and design

### Design

The study was qualitative and exploratory in nature.^18^

### Population and sampling

The study population consisted of level III student midwives at university, professional midwives at state and private hospitals and educators employed at a university and a college. The study was conducted at the university in Tshwane where the level III student midwives were registered for an undergraduate nursing programme. The target group of participants for level III student midwives was all the undergraduate full-time students who were registered for third-year Midwifery theoretical and practical subjects in 2007 – a total of 31.^4^ The second group of participants was the professional midwives who were employed by state and private health institutions in Tshwane where the undergraduate nursing students were placed for work integrated learning (WIL). The third group of participants was the educators teaching Midwifery in nursing colleges and universities in Tshwane.^25^

The sampling method for the level III student midwives and the professional midwives was convenient and purposive. The level III students were sampled as they were more informed about the topic under study as they had been instructed about the concept of caring, had passed midwifery II and were placed for WIL in the maternity sections of clinics and hospitals in Tshwane.

All the professional midwives who had been permanently employed for five years or more in clinics and hospitals in Tshwane were invited to participate in the study as they are expected to practice and have an in-depth knowledge of caring. A census sampling method was used for the educators as all those who were invited to take part agreed to participate in the study.

### Data-collection instruments

Self-report was the main data-collection method for all three groups of participants. Focus group sessions and focus group schedules were the method and instrument used for the student midwives and professional midwives, whilst narratives and the naive sketch form were used as method and instrument for educators.

The focus group schedule and the naive sketch form comprised two sections. Section A was dedicated to the demographic profile of the participants, such as age and years of work experience. Section B of the focus group schedule posed a central question, namely:

‘From your own perspective, please tell me the meaning of caring in midwifery clinical practice.’

Section B of the naive sketch form asked the educators to narrate the meaning of caring:

‘As a midwifery educator, please tell me the meaning of caring in midwifery clinical practice.’

### Pretesting of the instruments

Pretesting of the focus group interview was conducted with a group of six level III student midwives who were not included in the study. It was confirmed that the interview lasted 45 to 60 minutes and the central question was rephrased. The instrument for the professional midwives was pretested on four professional midwives working at a hospital which was not included in the study, and there was no need to adjust any part of the instrument. The naïve sketch was pretested at a university not included in the study; years of experience as an educator was added to the tool.

### Data collection

The data were collected by the first author, who used English as language of communication. The study purpose and data collection process were explained to the student midwives in a class-room at the university where they were registered in the undergraduate programme. Those who agreed to participate signed a consent form. Arrangements were made with the Head of the Nursing Department of the university to access the student midwives. Appointments were made with them a week before the focus group session took place. Two focus groups were conducted with the level III student midwives. A third focus group was conducted but no new information emerged.

The focus group was conducted in the skills laboratory, with the assistance of the moderator, a psychiatric nurse who had interviewing skills. Three focus group interviews comprising six to seven student midwives were planned for data collection purposes. The seating arrangement was in the form of a circle in order to facilitate eye contact in the focus group. A tape recorder was used to record the interviews after the student midwives granted permission to record the interview. The researcher was the student midwives’ lecturer hence the student midwives were called by their names during the interview; however, pseudonyms were used during transcription of the data.

The Department of Health, managers of the hospitals, clinics, and the University Ethics committee were consulted and permission to interview the professional midwives and the educators respectively was obtained. Prior arrangement was made with the professional midwives to conduct the focus group and a briefing about the nature of the study was made. Professional midwives were reminded of the focus group on the morning of the interview. On the day of the interview, the professional midwives were informed of the nature of the study and that a tape recorder was to be used. They were requested to sign a consent form to indicate their willingness to participate. Seventeen professional midwives were interviewed in two focus groups. The researcher conducted the focus group, whilst the moderator operated the tape recorder and took field notes. The focus group took place in the duty room, away from the rest of the personnel, in order to minimise disturbances. The process took about an hour.

Permission to conduct the study at the university and college to access the educators was granted. Also, arrangements were made with the Heads of Department at the college and university. Email addresses of the educators were obtained and the naïve sketch forms were dispatched to them; they were given a week to complete the form. Ten naïve sketch forms were sent to the educators; nine were completed and returned. Table 1 indicates the number of participants, data-collection methods, and the instruments used.

**TABLE 1:** Population, data-collection methods and instruments.

Population	Sample size	Data-collection method	Instrument
Students midwives	12	Two focus groups	Focus group schedule
Midwives	17	Two focus groups	Focus group schedule
Educators	9	Narratives	Naïve sketch form

The total sample realised for the study was 38: two focus groups of student midwives with six participants each, eight and nine participants in the first and second group of professional midwives respectively, and nine educators.

### Data analysis

Data analysis commenced as soon as the first interview was conducted and the first naïve sketch was received. The content analysis approach was taken using open coding according to Tesch’s approach.^26^ Responses from the interviews were transcribed verbatim and later categorised into themes and subcategories. The findings from each of the three groups of participants were classified into themes, categories and subcategories.

### Trustworthiness

The principles of trustworthiness proposed by Lincoln and Guba^27^ were considered.

Credibility was ensured by the fact that the researcher had 10 years’ experience as a researcher, educator and midwife. As a result, she had a relationship of trust with the student midwives, educators in the midwifery field and midwives in the clinical areas. The researcher conducted the focus group amongst all three groups of participants in order to promote consistency using the designed tool. Furthermore, the researcher and the moderator each analysed the data separately, compared their findings, and reached an agreement regarding the meaning the participants ascribed to caring. This was done to increase the credibility of the findings.

Confirmability was ensured by conducting a member checking to confirm with the participants that the data recorded were the true reflection of the focus group session and the narratives.

Dependability was ensured by following the scientific process of conducting a research.

Transferability was ensured by clearly describing the context of the study so that the findings could be applied in similar settings.

## Ethical considerations

Approval to conduct the study was granted by the Research and Ethics Committee of the University (2006/10/025), the Gauteng Department of Health Research Unit, the heads of the institutions of learning and the managers of the different hospitals identified.^15^ Respect for persons, autonomy, beneficence, justice, confidentiality and anonymity principles were adhered to.^18^ In addition, the participants completed a written informed consent form to indicate their voluntary participation in the study. The participants were further informed that they were allowed to terminate their participation without facing any penalty.

## Findings of the study

### Demographic profile of the participants

Table 2 shows the demographic profile of the three groups of participants.

**TABLE 2:** Demographic profile of the participants (n = 38).

Criteria	Student midwives	*n*	Professional midwives	*n*	Educators	*n*
Age	18–21	7	21–30	3	31–40	3
	22–30	5	31–50	11	41–50	4
	> 30	0	> 50	3	> 50	2
South African languages†	Afrikaans	2	Afrikaans	4	Setswana	6
	Sesotho	1	Sesotho	4	Sesotho	2
	TshiVenda	3	TshiVenda	3	isiTsonga	1
	isiTsonga isiNdebele		isiTsongaisiNdebele			
	isiXhosa isiSwati English	3	isiXhosa	3	-	0
			isiSwati			
			English			
	Sepedi Setswana	2	SetswanaSepediisiZulu	3		0
Foreign languages	Foreign	11	-	0	-	0
Gender	Female	10	Female	17	Female	9
	Male	2	Male	0	Male	0
Years of work experience	Not applicable to student midwives		< 5	5	5–10	0
			6–10	3	11–15	1
			11–20	6	16–20	7
			> 21	3	> 21	1
**Total**	**12**		**17**		**9**	

† South African languages: Sepedi, TshiVenda, Sesotho, Tsonga, Setswana, isiZulu, isiXhosa, isiNdebele, English, isiSwati and Afrikaans.

The 12 undergraduate student midwives’ ages ranged from 18 to 30 years; 7 of the 12 were in the age group 18 to 21 years. There is an age gap of more than 10 years between the youngest student midwife and the youngest educator, and a further age gap of 10 years between the youngest educator and the youngest midwife. Eleven out of 12 student midwives spoke 1 of the 11 South African official languages; two of this group were Afrikaans speaking whilst one spoke a foreign language.

Eleven of the 17 professional midwives were between 31 and 50 years old whilst 4 of the 9 educators were between 41 and 50 years old. Seven educators had 16 to 20 years’ of work experience. Sixteen midwives stated that they enjoyed working in midwifery clinical practice.

### Themes

Three themes emerged from the data (Table 3). Both negative and positive sentiments were expressed; examples of these are provided under each theme.

**TABLE 3:** Findings of the study.

Theme	Student midwives	Professional midwives	Educators
Caring and competence as the basis of nursing	Realising the basic needs of the patient	Being dedicated to the work	Ensuring that one remains competent
Compassion and support	Being there for somebody when the need arises	Assisting the person to maintain his physical, mental and emotional aspect of health	Identifying and attending to student midwives’or patients’ needs
Respect for humankind	Showing respect	Giving respect to the patients	Respecting individuality

#### Caring and competence as the basis of nursing

According to the some participants, preparing the favourable conducive environment for care is perceived as caring. The student midwives expressed caring as when one gives the best care to patients using expert knowledge. They described the confidence the professional midwives had when assisting them during a delivery and how they felt comfortable during a birth as competence and caring. The educators expressed that rendering service based on individual needs, respect, affection, and dignity reflect caring whilst the midwives perceived dedication to one’s work as competency and caring. In addition, the midwives ascribed caring to instances where there was integration of cultural beliefs, practices and values in the scientific assessment and care of their patients.

#### Realities related to caring and competence as the basis of nursing

The realities of this category are both positive and negative as experienced by the student midwives, professional midwives and educators. Students observed behaviour that demonstrated competency as well as a lack of competency. The student midwives expressed that they were supported and mentored during their WIL experience whilst some of them experienced negative realities.

The midwives stated that there was uncooperativeness and unfriendliness amongst some of their colleagues which resulted in uncaring and incompetent behaviour. Furthermore, midwives expressed lack of trust and confidence as a result of criticism from colleagues. As a result, there were no role models for newly qualified and student midwives. The educators stated that the ability to make accurate assessments and make good decisions to meet the students’ needs demonstrate caring and competence.

Participants expressed the following positive realities:

‘The midwife at the clinic was professional and passionate about her work. Sister made me and the patient … comfortable.’ (S5, 21 years)

‘A positive welcome of the patients and family in the unit and exercising patience demonstrate caring. Being knowledgeable about the work.’ (P2, 30 years)

‘Ensuring that one remains competent with the profession’s current body of knowledge.’ (E1, 40 years)

However, negative realities were also expressed:

‘No orientation to the unit or supervision of the students was done. This resulted in me becoming frustrated and wishing to take my bag and going home on that day.’ (S1, 19 years)

‘Patients suffer because of lack of teamwork. Some of the midwives refuse to give patients attention and treatments.’ (P4, 23 years)

‘Providing the student midwives with physical, psychological and social support. Caring means allowing the students to reflect on their learning and facilitation.’ (E6, 47 years)

#### Compassion and support to others in need

According to some participants, caring means giving people comfort. Caring was perceived as understanding someone’s difficult situation. According to the student midwives, caring means to ‘be there’ for somebody when the need arises, and nurturing is a positive way of helping someone. The professional midwives described caring as unconditional support of the woman and her family. The educators referred to caring as assisting the student midwives who are not performing well in class, following up with them and providing remedial action. The educators expressed that attending to student midwives’ or patients’ needs demonstrate caring.

#### Realities related to compassion and support to others in need

Student midwives experienced mostly negative realities related to compassion and support to others in need. Student midwives observed that the behaviours of some of the professional midwives were disturbing, citing some incidents that occurred in their presence as uncaring. In one incident, the student midwives work schedule was amended without informing them of it. When student midwives came to work the following day, they were sent back home as they were not supposed to be on duty according to the changed duty list. The student midwives regarded that as insensitivity and lack of positive regard.

The midwives also reported that they observed uncaring behaviours amongst their own colleagues. From the professional midwives’ perspectives, their colleagues did not care for one another as colleagues; the outcome is an environment which is demotivating, resulting in the deterioration of work quality. The educators expressed ‘academic’ and physical definitions of the reality of caring. They demonstrated that they have been taught the meaning of caring. The following definitions of caring were expressed:

‘Caring means assisting someone without expecting reward. A partner or a family member was allowed into the delivery room to provide support to the woman. This I think was real caring.’ (S11, 30 years)

‘Administration of analgesia to ensure comfort when someone is in pain, means caring.’ (P3, 51 years)

‘Giving your time, service and holistic attention to others, being interested in the wellbeing of others, being empathetic, means caring.’ (E9, 31 years)

The participants also experienced negative realities:

‘A patient who had miscarriage earlier in the year was scolded for being pregnant twice in one year. The patient was so embarrassed.’ (S10, 25 years)

‘As a result of an uncaring environment, the turnover of the midwives is high and some of the midwives engage in overtime encounters in an attempt to acquire financial and job satisfaction elsewhere.’ (P3, 49 years)

#### Respect for humankind

According to the participants’ perceptions, caring is demonstrated when individuals, their families and their beliefs are respected. The student midwives and professional midwives stated that one should show respect or give respect to others whilst the educators mentioned that respecting the other person’s individuality, norms, values and cultural practices demonstrates caring. Some of the student midwives observed disrespectful behaviour towards patients and other professionals by professional midwives, whilst the midwives articulated that respect shown to patients and other professionals was inconsistent.

#### Realities related to respect for humankind

The student midwives stated that some of the professional midwives were seen mishandling patients without talking to them. Some professional midwives claimed that respect by some of their colleagues is inconsistent. According to them, there often was infighting about allocation of work, which indicates disrespect of authority. The professional midwives mentioned that some of their colleagues undermined the decisions taken by others. This state of affairs negatively affected the quality of patient care. The educators’ view was that respecting the patient’s decision-making abilities and individuality means caring:

‘Showing respect and unconditional focus directed at the person means caring.’ (S7, 25 years)

‘Respect to the patients’ wishes in the management and care shows caring. Treating the patient as unique individual whilst providing privacy and exercising confidentiality.’ (P3, 37 years)

‘Providing respectful treatments, being gentle, nurturing, respecting the family and their beliefs.’ (E8, 33 years)

## Discussion

From the findings about the meaning of caring in midwifery practice, it is apparent that the student midwives, professional midwives and educators have almost the same interpretation and understanding of caring at a cognitive level. The student midwives, however, emphasised emotional presence and showing respect as expressions of caring. These two aspects denote that the student midwives knew that caring is both instrumental and expressive, as postulated by Woodward^29^ and Mulaudzi^30^ et al. A study by Murphy^31^ et al. in the United Kingdom found that because of an uncaring clinical environment, the expressive caring value of student nurses were gradually lost over the period of training. In the current study, however, the student midwives were not discouraged or preoccupied by an uncaring midwifery clinical environment.

Smith et al.^32^ stated in their study about caring in nursing that no other profession is as totally concerned with caring behaviours, caring processes and caring relationship as nursing and midwifery. In the current study, the professional midwives and educators indicated the same – that caring, competence and compassion constitute caring in midwifery clinical practice. Roach^10^ asserts that compassion without competence in nursing and midwifery is meaningless.

The professional midwives stated that teamwork is poor and some professional midwives have no respect for authority. Disconcertingly, poor teamwork has a negative influence on quality of care and patient outcomes.^33^ In a study by Smith et al.^32^ the registered nurses were asked to rank several meanings of caring in order of importance. Some of the meanings were attentive listening, comforting, honesty, patience, responsibility, providing information for decision making, touch, sensitivity, respect, and individuality. The participants in the current study referred to a few of these, namely respect, individuality and comforting. Once again, this agrees with Roach’s definition of caring, which is being compassionate.^10^

Caring is unique to each individual and it the right of everyone to define this concept according to psychological, cultural, social and spiritual needs.^9^ In the current study, all the participants defined caring as being compassionate and showing respect; however, it seems the educators put more emphasis on the instrumental role of caring. The educators referred to ‘identifying’ and attending to the needs of patients or students and ‘respecting individuality’ as the meaning of caring. Therefore, they were emphasising the physical aspect of caring more than the emotional. The definition is related to Watson’s clinical caritas of caring, which states that caring is being considerate and assisting someone with basic needs.^13^ It seems teaching caring has also been affected by the political, social and technological changes in the profession, which resulted in the profession losing its humanness, as stated by Watson.^13^ There seems to be a gap in the process of professional growth on caring which shows a difference between cognitive assertions of the concept, the psychomotor and the affective behaviour.

Some participants mentioned that respecting patients’ privacy, individuality and wishes in their management and care constitute caring.^4^ This is an important aspect in the management of women in labour, as failure to involve women in their management could increase catecholamine in the body as a response to anxiety, pain and fear.^34^ Indeed, this will result in prolonged labour and hampers the woman’s coping mechanism regarding the pain of labour.^34^ Watson supports this aspect and mentions that caring includes promotion of sensitivity to oneself and going beyond your ego self.^13^,^31^,^32^ With this study, the researchers aimed to emphasise that humanness is the foundation of midwifery and to encourage the interconnectedness of the one providing care and the one being cared for.

## Limitations

Data were collected from student midwives, professional midwives and educators only; a possible limitation of the study is that patients were not asked to define caring. This might have enriched the data.

## Recommendations

The midwifery and nursing professions should have frequent in-service training on caring and include caring processes and relationships during clinical practice and at training institutions. The caring curriculum should run through the undergraduate nursing programme, the attributes of caring highlighted throughout and emphasised more as students become more senior.

## Conclusion

In this study, student midwives’, professional midwives’ and educators’ meaning of caring were explored. The participants described caring as respect, compassion and competence – the basis for nursing. The training of students should expose them to authentic learning opportunities about caring. Furthermore, novice midwives should be exposed to positive caring role models.
